# Decomposition of Fuzzy Soft Sets with Finite Value Spaces

**DOI:** 10.1155/2014/902687

**Published:** 2014-01-12

**Authors:** Feng Feng, Hamido Fujita, Young Bae Jun, Madad Khan

**Affiliations:** ^1^Department of Applied Mathematics, School of Science, Xi'an University of Posts and Telecommunications, Xi'an 710121, China; ^2^Faculty of Software and Information Science, Iwate Prefectural University, Iwate 020-0193, Japan; ^3^Department of Mathematics Education (and RINS), Gyeongsang National University, Jinju 660-701, Republic of Korea; ^4^Department of Mathematics, COMSATS Institute of Information Technology, Abbottabad 22060, Pakistan

## Abstract

The notion of fuzzy soft sets is a hybrid soft computing model that integrates both gradualness and parameterization methods in harmony to deal with uncertainty. The decomposition of fuzzy soft sets is of great importance in both theory and practical applications with regard to decision making under uncertainty. This study aims to explore decomposition of fuzzy soft sets with finite value spaces. Scalar uni-product and int-product operations of fuzzy soft sets are introduced and some related properties are investigated. Using *t*-level soft sets, we define level equivalent relations and show that the quotient structure of the unit interval induced by level equivalent relations is isomorphic to the lattice consisting of all *t*-level soft sets of a given fuzzy soft set. We also introduce the concepts of crucial threshold values and complete threshold sets. Finally, some decomposition theorems for fuzzy soft sets with finite value spaces are established, illustrated by an example concerning the classification and rating of multimedia cell phones. The obtained results extend some classical decomposition theorems of fuzzy sets, since every fuzzy set can be viewed as a fuzzy soft set with a single parameter.

## 1. Introduction

With the development of modern science and technology, modelling various uncertainties has become an important task for a wide range of applications including data mining, pattern recognition, decision analysis, machine learning, and intelligent systems. The concept of uncertainty is too complicated to be captured within a single mathematical framework. In response to this situation, a number of approaches including probability theory, fuzzy sets [1], and rough sets [2] have been developed. Generally speaking, these theories deal with uncertainty from distinct angle of views, namely, randomness, gradualness, and granulation, respectively. Molodtsov's soft set theory [3] is a relatively new mathematical model for coping with uncertainty from a parametrization point of view. Zhu and Wen [4] redefined and improved some set-theoretic operations of soft sets that inherit all basic properties of operations on classical sets. Maji et al. [5] initiated the notion of fuzzy soft sets, which is a hybrid soft computing model in which the viewpoints of gradualness and parametrization for dealing with uncertainty are combined effectively. Majumdar and Samanta [6] further considered generalized fuzzy soft sets and applied them to decision making and medical diagnosis problems. Yang et al. [7] generalized fuzzy soft sets to interval-valued fuzzy soft sets. Up to now, fuzzy soft sets have proven to be useful in various fields such as flood alarm prediction [8], medical diagnosis [9], combined forecasting [10], supply chains risk management [11], topology [12], decision making under uncertainty [13–15], and algebraic structures [16–24].

The notion of level soft sets plays a crucial role in solving uncertain decision-making problems based on fuzzy soft sets [13]. In particular, it is important to figure out how many different *t*-level soft sets could be obtained from a given fuzzy soft set by choosing distinct threshold values *t* ∈ [0,1]. On the other hand, it is well known that decomposition theorems are of great theoretical importance in exploring various types of fuzzy structures. Thus decomposition of fuzzy soft sets is a topic of both theoretical and practical value. Motivated by this consideration, Feng et al. investigated some basic properties of level soft sets based on variable thresholds and obtained some decomposition theorems of fuzzy soft sets by considering variable thresholds [25]. Moreover, Feng and Pedrycz [26] carried out a detailed research on scalar products and decomposition of fuzzy soft sets. Particularly, They have shown that scalar product operations can be regarded as semimodule actions and algebraic structures like ordered idempotent semimodules of fuzzy soft sets over ordered semirings can be constructed [26]. In most real applications, especially those involving the use of computers and programs, we only need to consider a finite universe of discourse associated with finite number of parameters. Consequently, in this study, we shall follow the research line above and concentrate on decomposition of fuzzy soft sets with finite value spaces.

The remainder of this paper is organized as follows.  Section 2 first recalls some basic notions concerning fuzzy sets, soft sets, and fuzzy soft sets.  Section 3 mainly introduces *t*-level soft sets and scalar uniproduct operations of fuzzy soft sets. Then we explore some lattice structures associated with level soft sets in detail. In Section 5, we consider the decomposition of fuzzy soft sets with finite value spaces and establish some useful decomposition theorems, supported by illustrative examples. Finally, the last section summarizes the study and suggests possible directions for future work.

## 2. Preliminaries

In this section, we briefly review some basic concepts concerning fuzzy sets, soft sets, and fuzzy soft sets, respectively.

### 2.1. Fuzzy Sets

The theory of fuzzy sets [1] provides an appropriate framework for representing and processing vague concepts by admitting a notion of a partial membership. Recall that a fuzzy set *μ* in a universe *U* is defined by (and usually identified with) its *membership function* *μ* : *U* → [0,1]. For *x* ∈ *U*, the membership value *μ*(*x*) essentially specifies the degree to which *x* ∈ *U* belongs to the fuzzy set *μ*. By *μ*⊆*ν*, we mean that *μ*(*x*) ≤ *ν*(*x*) for all *x* ∈ *U*. Clearly *μ* = *ν* if *μ*⊆*ν* and *ν*⊆*μ*. That is, *μ*(*x*) = *ν*(*x*) for all *x* ∈ *U*. Let $\hat{t}$ denote the fuzzy set in *U* with a constant membership value *t* ∈ [0,1]; that is, $\hat{t}(x)=t$ for all *x* ∈ *U*. In what follows, we denote by *ℱ*(*U*) the set of all fuzzy sets in *U*.

Let *t* ∈ [0,1] and *μ* ∈ *ℱ*(*U*). Recall that the *scalar product* of *t* and *μ* is a fuzzy set *tμ* ∈ *ℱ*(*U*) defined by *tμ*(*x*) = *t*∧*μ*(*x*) for all *x* ∈ *U*. There are different definitions for fuzzy set operations. With the min-max system proposed by Zadeh, fuzzy set *intersection, union,* and *complement* are defined as follows:

- (*μ*∩*ν*)(*x*) = min{*μ*(*x*), *ν*(*x*)},
- (*μ* ∪ *ν*)(*x*) = max{*μ*(*x*), *ν*(*x*)},
- *μ* ^*c*^(*x*) = 1 − *μ*(*x*),

where *μ*, *ν* ∈ *ℱ*(*U*) and *x* ∈ *U*.

### 2.2. Soft Sets

Soft set theory was proposed by Molodtsov [3] in 1999, which provides a general mechanism for uncertainty modelling in a wide variety of applications. Let *U* be the universe of discourse and let *E* be the universe of all possible parameters related to the objects in *U*. In most cases, parameters are considered to be attributes, characteristics, or properties of objects in *U*. The pair (*U*, *E*) is also known as a *soft universe*. The power set of *U* is denoted by *𝒫*(*U*).

**Definition 1 (see [3])**

A pair *𝔖* = (*F*, *A*) is called a *soft set* over *U*, where *A*⊆*E* and *F* : *A* → *𝒫*(*U*) is a set-valued mapping, called the *approximate function* of the soft set *𝔖*.

By means of parametrization, a soft set gives a series of approximate descriptions of a complicated object being perceived from distinct aspects. For each parameter *ϵ* ∈ *A*, the subset *F*(*ϵ*)⊆*U* is known as the set of *ϵ-approximate elements* [3]. It is worth noting that *F*(*ϵ*) may be arbitrary: some of them may be empty, and some may have nonempty intersections. In what follows, the collection of all soft sets over *U* with parameter sets contained in *E* is denoted by *𝒮* ^*E*^(*U*). Moreover, we denote by *𝒮*_*A*_(*U*) the collection of all soft sets over *U* with a fixed parameter set *A*⊆*E*.

Maji et al. [27] introduced the concept of soft M-subsets and soft M-equal relations in the following manner.

**Definition 2 (see [27])**

Let (*F*, *A*) and (*G*, *B*) be two soft sets over *U*. Then (*F*, *A*) is called a *soft M-subset *of (*G*, *B*), denoted by  $\left(F,A){\tilde{\subseteq }}_{M}(G,B\right)$, if *A*⊆*B* and *F*(*a*) = *G*(*a*) (i.e., *F*(*a*) and *G*(*a*) are identical approximations) for all *a* ∈ *A*. Two soft sets (*F*, *A*) and (*G*, *B*) are said to be *soft M-equal*, denoted by  (*F*, *A*) =_*M*_(*G*, *B*), if $\left(F,A\right){\tilde{\subseteq }}_{M}\left(G,B\right)$ and $\left(G,B\right){\tilde{\subseteq }}_{M}\left(F,A\right)$.

Another different type of soft subsets and soft equal relations can be defined as follows.

**Definition 3 (see [28])**

Let (*F*, *A*) and (*G*, *B*) be two soft sets over *U*. Then (*F*, *A*) is called a *soft F-subset *of (*G*, *B*), denoted by  $\left(F,A){\tilde{\subseteq }}_{F}(G,B\right)$, if *A*⊆*B* and *F*(*a*)⊆*G*(*a*) for all *a* ∈ *A*. Two soft sets (*F*, *A*) and (*G*, *B*) are said to be *soft F-equal*, denoted by  (*F*, *A*) =_*F*_(*G*, *B*), if $\left(F,A\right){\tilde{\subseteq }}_{F}\left(G,B\right)$ and $\left(G,B\right){\tilde{\subseteq }}_{F}\left(F,A\right)$.

It is easy to see that, for two soft sets *𝔖* = (*F*, *A*) and *𝔗* = (*G*, *B*), if *𝔖* is a soft M-subset of *𝔗*, then *𝔖* is also a soft F-subset of *𝔗*. However, the converse may not be true (see Example  2.6 in [29]). As shown in [29], the soft equal relations =_*M*_ and =_*F*_ coincide with each other. Hence in what follows, we just call them soft equal relations and simply write = instead of =_*M*_ or =_*F*_ unless stated otherwise.

**Definition 4 (see [30])**

Let *𝔖* = (*F*, *A*) be a soft set over *U*. Then

- *𝔖* is called a *relative null soft set* (with respect to the parameter set *A*), denoted by Φ_*A*_, if *F*(*a*) = *∅* for all *a* ∈ *A*;
- *𝔖* is called a *relative whole soft set* (with respect to the parameter set *A*), denoted by *𝔘*_*A*_, if *F*(*a*) = *U* for all *a* ∈ *A*.

### 2.3. Fuzzy Soft Sets

By combining fuzzy sets with soft sets, Maji et al. [5] initiated a hybrid soft computing model called fuzzy soft sets as follows.

**Definition 5 (see [5])**

A pair $𝔖=(\tilde{F},A)$ is called a *fuzzy soft set *over *U*, where *A*⊆*E* and $\tilde{F}$ is a mapping given by $\tilde{F}:A\to ℱ(U)$.

Conventionally, the mapping $\tilde{F}:A\to ℱ(U)$ is referred to as the *approximate function* of the fuzzy soft set $\left(\tilde{F},A\right)$. It is easy to see that fuzzy soft sets extend Molodtsov's soft sets by substituting fuzzy subsets for crisp subsets. Note also that a fuzzy set could be viewed as a fuzzy soft set whose parameter set reduces to a singleton. This means that fuzzy soft sets can be seen as a parameterized extension of fuzzy sets and it can be used to model those complicate fuzzy concepts which cannot be described using a single fuzzy set or simply by the intersection of some fuzzy sets.

As in the case of soft sets, different types of fuzzy soft subsets can be introduced. Given two fuzzy soft sets $\left(\tilde{F},A\right)$ and $\left(\tilde{G},B\right)$ over *U*, we say that $\left(\tilde{F},A\right)$ is a *fuzzy soft F-subset *of $\left(\tilde{G},B\right)$, denoted by $\left(\tilde{F},A)\;{\tilde{\subseteq }}_{F}\;(\tilde{G},B\right)$, if *A*⊆*B* and $\tilde{F}(a)$ is a fuzzy subset of $\tilde{G}(a)$ for all *a* ∈ *A*. On the other hand, $\left(\tilde{F},A\right)$ is called a *fuzzy soft M-subset *of $\left(\tilde{G},B\right)$, denoted by $\left(\tilde{F},A)\;{\tilde{\subseteq }}_{M}\;(\tilde{G},B\right)$, if *A*⊆*B* and $\tilde{F}(a)=\tilde{G}(a)$ for all *a* ∈ *A*. Two fuzzy soft sets $\left(\tilde{F},A\right)$ and $\left(\tilde{G},B\right)$ over *U* are said to be *fuzzy soft equal* if they are identical; that is, *A* = *B* and $\tilde{F}(a)=\tilde{G}(a)$ for all *a* ∈ *A*. This is denoted by $\left(\tilde{F},A)=(\tilde{G},B\right)$.

**Definition 6**

Let $𝔖=(\tilde{F},A)$ be a fuzzy soft set over *U* and *t* ∈ [0,1]. Then *𝔖* is called a *t-constant fuzzy soft set*, denoted by ${\tilde{ℭ}}_{A}^{t}$, if $\tilde{F}(a)=\hat{t}$ for all *a* ∈ *A*.

In particular, ${\tilde{ℭ}}_{A}^{0}$ and ${\tilde{ℭ}}_{A}^{1}$ are called the *relative null fuzzy soft set* and *relative whole fuzzy soft setry 23*

(with respect to the parameter set *A*), respectively.

**Definition 7 (see [25])**

Let $\left(\tilde{F},A\right)$ and $\left(\tilde{G},B\right)$ be two fuzzy soft sets over *U*.

(1) The *extended union* of $\left(\tilde{F},A\right)$ and $\left(\tilde{G},B\right)$ is defined as the fuzzy soft set $\left(\tilde{H},C\right)=\left(\tilde{F},A\right)\cup_{ℰ}\left(\tilde{G},B\right)$, where *C* = *A* ∪ *B* and for all *c* ∈ *C*,
  (1) $$\begin{array}{c} \tilde{H}\left(c\right)=\{\begin{array}{cc} \tilde{F}\left(c\right), & \text{if}\;\;c\in A\setminus B,\\ \tilde{G}\left(c\right), & \text{if}\;\;c\in B\setminus A,\\ \tilde{F}\left(c\right)\cup \tilde{G}\left(c\right), & \text{if}\;\;c\in A\cap B. \end{array} \end{array}$$
(2) The *extended intersection* of $\left(\tilde{F},A\right)$ and $\left(\tilde{G},B\right)$ is defined as the fuzzy soft set $\left(\tilde{H},C\right)=\left(\tilde{F},A\right)\cap_{ℰ}\left(\tilde{G},B\right)$, where *C* = *A* ∪ *B* and for all *c* ∈ *C*,
  (2) $$\begin{array}{c} \tilde{H}\left(c\right)=\{\begin{array}{cc} \tilde{F}\left(c\right), & \text{if}\;\;c\in A\setminus B,\\ \tilde{G}\left(c\right), & \text{if}\;\;c\in B\setminus A,\\ \tilde{F}\left(c\right)\cap \tilde{G}\left(c\right), & \text{if}\;\;c\in A\cap B. \end{array} \end{array}$$
(3) The *restricted intersection* of $\left(\tilde{F},A\right)$ and $\left(\tilde{G},B\right)$ is defined as the fuzzy soft set $\left(\tilde{H},C\right)=\left(\tilde{F},A\right)\cap_{ℛ}\left(\tilde{G},B\right)$, where *C* = *A*∩*B* ≠ *∅* and $\tilde{H}\left(c\right)=\tilde{F}\left(c\right)\cap \tilde{G}\left(c\right)$ for all *c* ∈ *C*.
(4) The *restricted union* of $\left(\tilde{F},A\right)$ and $\left(\tilde{G},B\right)$ is defined as the fuzzy soft set $\left(\tilde{H},C\right)=\left(\tilde{F},A\right)\cup_{ℛ}\left(\tilde{G},B\right)$, where *C* = *A*∩*B* ≠ *∅* and $\tilde{H}\left(c\right)=\tilde{F}\left(c\right)\cup \tilde{G}\left(c\right)$ for all *c* ∈ *C*.

In what follows, the collection of all fuzzy soft sets over *U* with parameter sets contained in *E* is denoted by *ℱ𝒮* ^*E*^(*U*). Taking any parameter set *A*⊆*E*, one can consider the collection consisting of all fuzzy soft sets over *U* with the fixed parameter set *A*, which is denoted by *ℱ𝒮*_*A*_(*U*). The following result can easily be obtained using the above definitions.

**Proposition 8**

Let $\left(\tilde{F},A\right)$ and $\left(\tilde{G},A\right)$ be two fuzzy soft sets over *U*. Then

1. $\left(\tilde{F},A\right)\cup_{ℰ}\left(\tilde{G},A\right)=\left(\tilde{F},A\right)\cup_{ℛ}\left(\tilde{G},A\right)$;
2. $\left(\tilde{F},A\right)\cap_{ℰ}\left(\tilde{G},A\right)=\left(\tilde{F},A\right)\cap_{ℛ}\left(\tilde{G},A\right)$.

The first part of the above assertion states that, for fuzzy soft sets in *ℱ𝒮*_*A*_(*U*), the extended union ∪_*ℰ*_ coincides with the restricted union ∪_*ℛ*_. That is, the two soft union operations ∪_*ℛ*_ and ∪_*ℰ*_ will always lead to the same results when considering fuzzy soft sets with the same set of parameters. Thus in this case, we shall use a uniform notation $\tilde{\cup }$ representing both ∪_*ℛ*_ and ∪_*ℰ*_. Analogously ∩_*ℛ*_ and ∩_*ℰ*_ will be simply denoted by $\tilde{\cap }$ if the two operations coincide with each other.

Now we illustrate the notion of fuzzy soft sets by an example as follows.

**Example 9**

Suppose that there are six cell phones under consideration

(3) $$\begin{array}{c} U=\left\{p_{1},p_{2},p_{3},p_{4},p_{5},p_{6}\right\}. \end{array}$$

The set of parameters is given by *E* = {*e*_1_, *e*_2_, *e*_3_, *e*_4_, *e*_5_, *e*_6_, *e*_7_, *e*_8_}, where *e*_*i*_, respectively, stand for “high quality of voice call,” “stylish design,” “friendly user interface,” “wonderful MP3/MP4 playback,” “low price,” “high resolution camera,” “popular brand” and “large screen”.

Now, let *A* = {*e*_2_, *e*_4_, *e*_5_, *e*_6_, *e*_8_}⊆*E* consist of some crucial features for describing “attractive multimedia cell phones.” We can arrange an expert group to evaluate these cell phones and the available information on these mobile phones can be formulated as a fuzzy soft set $𝔖=(\tilde{F},A)$. It provides a mathematical representation of the complicate fuzzy concept, called “attractive multimedia cell phones” in daily languages.  Table 1 gives the tabular representation of the fuzzy soft set $𝔖=(\tilde{F},A)$.

Using this illustrative example, we can observe that some fuzzy concepts in the real world are so complicated that they can hardly be described using a single fuzzy set or simply the intersection of some fuzzy sets. Alternatively, these complicate fuzzy concepts can jointly be represented as a family of fuzzy sets organized by some useful parameters like those we list above for describing “attractive multimedia cell phones.” Based on the viewpoint of parametrization, each fuzzy set in a fuzzy soft set only produces an approximate (or partial) description of a complicated fuzzy concept, while the fuzzy soft set as a whole gives a complete representation.

## 3. Level Soft Sets and Scalar Uni-Products

To solve decision-making problems based on fuzzy soft sets, Feng et al. [13] introduced the following notion called *t*-level soft sets of fuzzy soft sets.

**Definition 10 (see [13])**

Let *t* ∈ [0,1] and $𝔖=(\tilde{F},A)$ be a fuzzy soft set over *U*. The *t-level soft set* of the fuzzy soft set *𝔖* is a crisp soft set *L*(*𝔖*; *t*) = (*F*_*t*_, *A*) over *U*, where

(4) $$\begin{array}{c} F_{t}\left(a\right)=\left\{x\in U:\tilde{F}\left(a\right)\left(x\right)\geq t\right\}, \end{array}$$

for all *a* ∈ *A*.

In the above definition, *t* ∈ [0,1] serves as a fixed threshold value on membership grades. In practical applications, these thresholds might be chosen by decision makers and represent the strength of their general requirements [13]. Some basic properties of *t*-level soft sets have been investigated by Feng and Pedrycz [26]. Below, we list two results, which show that the structure of *t*-level soft sets is compatible with some basic algebraic operations of fuzzy soft sets.

**Proposition 11 (see [26])**

Let $𝔖=(\tilde{F},A)$ and $ℜ=(\tilde{G},B)$ be two fuzzy soft sets in *ℱ𝒮* ^*E*^(*U*). Then, for all *t* ∈ [0,1], one has

- *L*(*𝔖*∪_*ℰ*_ *ℜ*; *t*) = *L*(*𝔖*; *t*)∪_*ℰ*_ *L*(*ℜ*; *t*);
- *L*(*𝔖*∩_*ℰ*_ *ℜ*; *t*) = *L*(*𝔖*; *t*)∩_*ℰ*_ *L*(*ℜ*; *t*).

**Proposition 12 (see [26])**

Let $𝔖=(\tilde{F},A)$ and $ℜ=(\tilde{G},B)$ be two fuzzy soft sets in *ℱ𝒮* ^*E*^(*U*) with *A*∩*B* ≠ *∅*. Then for all *t* ∈ [0,1], one has

- *L*(*𝔖*∪_*ℛ*_ *ℜ*; *t*) = *L*(*𝔖*; *t*)∪_*ℛ*_ *L*(*ℜ*; *t*);
- *L*(*𝔖*∩_*ℛ*_ *ℜ*; *t*) = *L*(*𝔖*; *t*)∩_*ℛ*_ *L*(*ℜ*; *t*).

We also have considered the following scalar product of a scalar value *t* and a fuzzy soft set in [26].

**Definition 13**

Let *t* ∈ [0,1] and $S=(\tilde{F},A)\in ℱ{𝒮}^{E}(U)$. Then the *scalar product* of *t* and *S* is defined to be a fuzzy soft set $t⊙S=(\tilde{G},A)$ over *U*, such that

(5) $$\begin{array}{c} \tilde{G}\left(a\right)\left(x\right)=\left(t\tilde{F}\left(a\right)\right)\left(x\right)=t\wedge \tilde{F}\left(a\right)\left(x\right), \end{array}$$

where *a* ∈ *A*, *x* ∈ *U*.

By replacing a single value *t* with a set *J*⊆[0,1] of thresholds, we immediately obtain an extension of the above concept.

**Definition 14**

Let *J*⊆[0,1] and $S=(\tilde{F},A)\in ℱ{𝒮}^{E}(U)$. Then the *scalar uniproduct* of *J* and *S* is defined as

(6) $$\begin{array}{c} J{⊙}_{\cup }S=\tilde{\underset{t\in J}{⋃}\;}t⊙S. \end{array}$$

Clearly, if *J* = {*t*} is a singleton, then *J*⊙_∪_ *S* = *t*⊙*S*. In a dual way, we can also define the following operation.

**Definition 15**

Let *J*⊆[0,1] and $S=(\tilde{F},A)\in ℱ{𝒮}^{E}(U)$. Then the *scalar int-product* of *J* and *S* is defined as

(7) $$\begin{array}{c} J{⊙}_{\cap }S=\tilde{\underset{t\in J}{⋂}\;}t⊙S. \end{array}$$

For *J* = *∅*, we define $J{⊙}_{\cup }S=J{⊙}_{\cap }S={\tilde{ℭ}}_{A}^{0}$. The following results give some basic properties of scalar uniproduct operations. Dually, we can also investigate related properties of scalar int-product operations.

**Proposition 16**

Let *J*_1_, *J*_2_⊆[0,1] and *S* ∈ *ℱ𝒮* ^*E*^(*U*). If *J*_1_⊆*J*_2_, then one has $J_{1}{⊙}_{\cup }S{\tilde{\subseteq }}_{F}J_{2}{⊙}_{\cup }S$.

**Proof**

The proof is straightforward and thus omitted.

**Proposition 17**

Let *J*⊆[0,1] and *S*_1_, *S*_2_ ∈ *ℱ𝒮* ^*E*^(*U*). If $S_{1}{\tilde{\subseteq }}_{F}S_{2}$, then one has $J{⊙}_{\cup }S_{1}{\tilde{\subseteq }}_{F}J{⊙}_{\cup }S_{2}$.

**Proof**

The proof is straightforward and thus omitted.

**Proposition 18**

Let *J*⊆[0,1] and *S*_1_, *S*_2_ ∈ *ℱ𝒮* ^*E*^(*U*). If $S_{1}{\tilde{\subseteq }}_{M}S_{2}$, then one has $J{⊙}_{\cup }S_{1}{\tilde{\subseteq }}_{M}J{⊙}_{\cup }S_{2}$.

**Proof**

The proof is straightforward and thus omitted.

## 4. Lattice Structures Associated with *t*-Level Soft Sets

In this section, we begin with some basic notions in lattice theory and then concentrate on exploring some lattice structures associated with *t*-level soft sets of a given fuzzy soft set. From an algebraic point of view, a *lattice*  (*L*, ∨, ∧) is a nonempty set with two binary operations ∨ and ∧ such that

1. (*L*, ∨) and (*L*, ∧) are commutative semigroups;
2. *a*∧(*a*∨*b*) = *a* and *a*∨(*a*∧*b*) = *a* for all *a*, *b* ∈ *L*.

Let (*L*, ∨, ∧) be a lattice. For every *a* ∈ *L*, it is easy to see that

(8) $$\begin{array}{c} a\vee a=a\vee \left(a\wedge \left(a\vee a\right)\right)=a. \end{array}$$

Similarly, we can deduce *a*∧*a* = *a*∧(*a*∨(*a*∧*a*)) = *a*. Thus in a lattice *L*, two operations ∨ and ∧ are both idempotent. That is, (*L*, ∨) and (*L*, ∧) are two *semilattices*.

If a lattice *L* has identity elements with respect to both ∨ and ∧, then *L* is said to be *bounded*. Usually identity element of *L* with respect to operation ∨ is denoted by ⊥, whereas the identity with respect to ∧ is denoted by *⊤*. In other words, (*L*, ∨, ⊥) and (*L*, ∧, *⊤*) are two *monoids* if (*L*, ∨, ∧, ⊥, *⊤*) is a bounded lattice.

**Definition 19**

A lattice (*L*, ∨, ∧) is called a *distributive lattice* if

1. *a*∧(*b*∨*c*) = (*a*∧*b*)∨(*a*∧*c*);
2. *a*∨(*b*∧*c*) = (*a*∨*b*)∧(*a*∨*c*);

for all *a*, *b*, *c* ∈ *L*.

**Example 20**

Let us consider the unit interval [0,1]. For all *a*, *b* ∈ *L*, we denote max{*a*, *b*} and min{*a*, *b*} by *a*∨*b* and *a*∧*b*, respectively. Then, it is easy to verify that ([0,1], ∨, ∧) is a distributive lattice. Moreover, ([0,1], ∨, ∧) is a bounded lattice with ⊥  = 0 and *⊤* = 1.

**Proposition 21**

Let *J*⊆[0,1] and *S* ∈ *ℱ𝒮* ^*E*^(*U*). Then *J*⊙_∪_ *S* = (∨*J*)⊙*S*.

**Proof**

Let $S=(\tilde{F},A)$ be a fuzzy soft set over *U*. Note first that t* = ∨*J* indeed exists since ([0,1], ∨, ∧) is a complete lattice. For any *t* ∈ *J*, we write $t⊙S=({\tilde{G}}_{t},A)$. Let *a* ∈ *A* and *x* ∈ *U*. By Definition 13, we have

(9) $$\begin{array}{c} {\tilde{G}}_{t}\left(a\right)\left(x\right)=t\wedge \tilde{F}\left(a\right)\left(x\right). \end{array}$$

If we write $J{⊙}_{\cup }S=(\tilde{H},A)$, then by the complete distributive laws in the complete lattice ([0,1], ∨, ∧), we have

(10) H~(a)(x)=⋁t∈JG~t(a)(x)=⋁t∈J(t∧F~(a)(x))=(⋁t∈Jt)∧F~(a)(x)=t∗∧F~(a)(x).

This implies that *J*⊙_∪_ *S* = t*⊙*S* = (∨*J*)⊙*S*.

Now, we consider the collection of all fuzzy soft sets over *U* with a fixed parameter set *A*. This collection forms a lattice structure with respect to soft union and intersection operations as shown in [26].

**Theorem 22**

$\left(ℱ{𝒮}_{A}(U),\tilde{\cup },\tilde{\cap },{\tilde{ℭ}}_{A}^{0},{\tilde{ℭ}}_{A}^{1}\right)$ is a bounded distributive lattice.

**Proof**

Let $S_{k}=({\tilde{F}}_{k},A)\in ℱ{𝒮}_{A}(U)$ (*k* = 1,2, 3). Write $S_{1}\tilde{\cup }S_{2}=(\tilde{H},A)$ and $S_{2}\tilde{\cup }S_{1}=(\tilde{R},A)$. Then for all *t* ∈ *A*, we have

(11) $$\begin{array}{c} \tilde{H}\left(t\right)={\tilde{F}}_{1}\left(t\right)\cup {\tilde{F}}_{2}\left(t\right)={\tilde{F}}_{2}\left(t\right)\cup {\tilde{F}}_{1}\left(t\right)=\tilde{R}\left(t\right). \end{array}$$

This shows that the soft union operation of fuzzy soft sets is commutative. Moreover, we can prove that this operation is associative. By definition of the relative null fuzzy soft set ${\tilde{ℭ}}_{A}^{0}$, it is easy to see that $S_{1}\tilde{\cup }{\tilde{ℭ}}_{A}^{0}=S_{1}$. Hence $\left(ℱ{𝒮}_{A}(U),\tilde{\cup },{\tilde{ℭ}}_{A}^{0}\right)$ is a commutative monoid. Dually, we deduce that $\left(ℱ{𝒮}_{A}(U),\tilde{\cap },{\tilde{ℭ}}_{A}^{1}\right)$ is a commutative monoid. Next, let $S_{1}\tilde{\cap }(\tilde{H},A)=(\tilde{K},A)$. Then for all *t* ∈ *A*, we have

(12) $$\begin{array}{c} \tilde{K}\left(t\right)={\tilde{F}}_{1}\left(t\right)\cap \tilde{H}\left(t\right)={\tilde{F}}_{1}\left(t\right)\cap \left({\tilde{F}}_{1}\left(t\right)\cup {\tilde{F}}_{2}\left(t\right)\right)={\tilde{F}}_{1}\left(t\right), \end{array}$$

and so $S_{1}\tilde{\cap }(S_{1}\tilde{\cup }S_{2})=S_{1}$. In a similar fashion, we can deduce $S_{1}\tilde{\cup }(S_{1}\tilde{\cap }S_{2})=S_{1}$. Thus $\left(ℱ{𝒮}_{A}(U),\tilde{\cup },\tilde{\cap },{\tilde{ℭ}}_{A}^{0},{\tilde{ℭ}}_{A}^{1}\right)$ is a bounded lattice. In addition, we write $S_{1}\tilde{\cup }S_{3}=(\tilde{G},A)$ and $S_{2}\tilde{\cap }S_{3}=(\tilde{J},A)$. Then we have

(13) F~1(t)∪J~(t)=(F~1(t)∪F~2(t))∩(F~1(t)∪F~3(t))=H~(t)∩G~(t),

which implies that $S_{1}\tilde{\cup }(S_{2}\tilde{\cap }S_{3})=(S_{1}\tilde{\cup }S_{2})\tilde{\cap }(S_{1}\tilde{\cup }S_{3})$. Dually, we have $S_{1}\tilde{\cap }(S_{2}\tilde{\cup }S_{3})=(S_{1}\tilde{\cap }S_{2})\tilde{\cup }(S_{1}\tilde{\cap }S_{3})$. Therefore, $\left(ℱ{𝒮}_{A}(U),\tilde{\cup },\tilde{\cap },{\tilde{ℭ}}_{A}^{0},{\tilde{ℭ}}_{A}^{1}\right)$ is a bounded distributive lattice.

As an immediate consequence of the above theorem, we get the following result in [31].

**Corollary 23**

$\left({𝒮}_{A}(U),\tilde{\cup },\tilde{\cap },\Phi_{A},{𝔘}_{A}\right)$ is a bounded distributive lattice.

Let (*L*, ∨, ∧) be a lattice. A nonempty set *M*⊆*L* is called a *sublattice* of *L* if *a*∨*b* ∈ *M* and *a*∧*b* ∈ *M* for all *a*, *b* ∈ *M*.

**Proposition 24**

Let $𝔖=(\tilde{F},A)\in ℱ{𝒮}^{E}(U)$ and *s*, *t* ∈ [0,1]. Then

- $L(𝔖;s\vee t)=L(𝔖;s)\tilde{\cap }L(𝔖;t)$;
- $L(𝔖;s\wedge t)=L(𝔖;s)\tilde{\cup }L(𝔖;t)$.

**Proof**

We only show that (a) is valid and (b) can be proved in a similar way. Let *L*(*𝔖*; *s*) = (*F*_*s*_, *A*), *L*(*𝔖*; *t*) = (*F*_*t*_, *A*), and *L*(*𝔖*; *s*∨*t*) = (*F*_*s*∨*t*_, *A*). Also, write $L(𝔖;s)\tilde{\cap }L(𝔖;t)=(H,A)$. For all *e* ∈ *A* and *u* ∈ *U*, we have

(14) u∈H(e)⟺u∈Fs(e)∩Ft(e)⟺F~(e)(u)≥s∨t⟺u∈Fs∨t(e),

and so *H*(*e*) = *F*_*s*∨*t*_(*e*) for all *e* ∈ *A*. Thus $L(𝔖;s\vee t)=L(𝔖;s)\;\tilde{\cap }\;L(𝔖;t)$ as required.

Let $𝔖=(\tilde{F},A)$ be a fuzzy soft set over *U*. We denote the collection of all *t*-level soft sets of the fuzzy soft set *𝔖* by *ℒ*(*𝔖*) = {*L*(*𝔖*; *t*) : *t* ∈ [0,1]}. Clearly, *ℒ*(*𝔖*) is a nonempty subset of *𝒮*_*A*_(*U*) since *𝔘*_*A*_ = *L*(*𝔖*; 0). In addition, by Proposition 24, we can immediately deduce the following result.

**Theorem 25**

*ℒ*(*𝔖*) is a sublattice of the lattice $\left({𝒮}_{A}(U),\tilde{\cup },\tilde{\cap }\right)$.

**Definition 26**

Let *𝔖* ∈ *ℱ𝒮* ^*E*^(*U*) and *s*, *t* ∈ [0,1]. Then *s* and *t* are said to be *level equivalent*, denoted by *s*~_*𝔖*_ *t*, if *L*(*𝔖*; *s*) = *L*(*𝔖*; *t*).

**Definition 27**

Let *L*_1_ and *L*_2_ be two lattices. A mapping *ψ* : *L*_1_ → *L*_2_ is a *homomorphism* of lattices if *ψ*(*a*∨*b*) = *ψ*(*a*)∨*ψ*(*b*) and *ψ*(*a*∧*b*) = *ψ*(*a*)∧*ψ*(*b*) for all *a*, *b* ∈ *L*_1_.

A homomorphism *ψ* : *L*_1_ → *L*_2_ of lattices is called a *monomorphism* (resp., *epimorphism* and *isomorphism*) if *ψ* is injective (resp., surjective and bijective). If *ψ* : *L*_1_ → *L*_2_ is an isomorphism of lattices, then *L*_1_ and *L*_2_ are said to be *isomorphic*, written as *L*_1_≅*L*_2_.

**Definition 28**

Let *ψ* : *L*_1_ → *L*_2_ be a homomorphism of lattices. Then the *kernel* of *ψ* is a binary relation on *L*_1_ defined by

(15) $$\begin{array}{c} \ker\left(\psi \right)=\left\{\left(a,b\right)\in L_{1}\times L_{1}:\psi \left(a\right)=\psi \left(b\right)\right\}. \end{array}$$

It is easy to check that *ker*(*ψ*) is an equivalence relation on *L*_1_. As a direct consequence, we deduce that the level equivalent relation ~_*𝔖*_ defined above is an equivalence relation on the unit interval [0,1].

**Proposition 29**

Let *𝔖* be a fuzzy soft set over *U* and [0,1]^†^ = ([0,1], ∧, ∨). Then the mapping *ψ*_*𝔖*_ : [0,1] → *ℒ*(*𝔖*) given by *ψ*_*𝔖*_(*t*) = *L*(*𝔖*; *t*) is an epimorphism of lattices with *ker*(*ψ*_*𝔖*_) = ~_*𝔖*_.

**Proof**

Note first that from Example 20, ([0,1], ∨, ∧) is a distributive lattice. Dually, we deduce that [0,1]^†^ = ([0,1], ∧, ∨) is also a distributive lattice. Using the notations given above, Proposition 24 implies $\psi_{𝔖}(s\wedge t)=\psi_{𝔖}(s)\;\tilde{\cup }\;\psi_{𝔖}(t)$ and $\psi_{𝔖}(s\vee t)=\psi_{𝔖}(s)\;\tilde{\cap }\;\psi_{𝔖}(t)$. Also it is clear that the mapping *ψ*_*𝔖*_ : [0,1] → *ℒ*(*𝔖*) is surjective. Thus it is an epimorphism of lattices. Moreover, it follows from Definitions 26 and 28 that *ker*(*ψ*_*𝔖*_) = ~_*𝔖*_.

Now, let us consider the quotient set of the unit interval [0,1] with respect to the level equivalent relation ~_*𝔖*_. We can define two operations on the quotient set [0,1]/~_*𝔖*_ as follows:

- [*s*]_~_*𝔖*__⊓[*t*]_~_*𝔖*__ = [*s*∧*t*]_~_*𝔖*__,
- [*s*]_~_*𝔖*__⊔[*t*]_~_*𝔖*__ = [*s*∨*t*]_~_*𝔖*__,

where [*s*]_~_*𝔖*__ denotes the equivalence class of *s* ∈ [0,1] under ~_*𝔖*_.

It can be shown that [0,1]^†^/~_*𝔖*_ = ([0,1]/~_*𝔖*_, ⊓, ⊔) is a lattice. In addition, we have the following result.

**Theorem 30**

Let *𝔖* be a fuzzy soft set over *U* and [0,1]^†^ = ([0,1], ∧, ∨). Then ${\left[0,1\right]}^{†}/\sim_{𝔖}=\left(\left[0,1\right]/\sim_{𝔖},⊓,⊔\right)≅(ℒ(𝔖),\tilde{\cup },\tilde{\cap })$.

**Proof**

Define a mapping *φ* : [0,1]/~_*𝔖*_ → *ℒ*(*𝔖*) by

(16) $$\begin{array}{c} \varphi \left({\left[t\right]}_{\sim_{𝔖}}\right)=\psi_{𝔖}\left(t\right)=L\left(𝔖;t\right). \end{array}$$

By Proposition 29, the mapping *ψ*_*𝔖*_ : [0,1] → *ℒ*(*𝔖*) given by *ψ*_*𝔖*_(*t*) = *L*(*𝔖*; *t*) is an epimorphism of lattices. Thus *φ* is surjective. Also we have

(17) φ([s]~𝔖⊓[t]~𝔖)=φ([s∧t]~𝔖)=ψ𝔖(s∧t)=ψ𝔖(s)∪~ψ𝔖(t)=φ([s]~𝔖)∪~φ([t]~𝔖).

Similarly, we can get $\varphi ({\left[s\right]}_{\sim_{𝔖}}⊔{\left[t\right]}_{\sim_{𝔖}})=\varphi ({\left[s\right]}_{\sim_{𝔖}})\tilde{\cap }\varphi ({\left[t\right]}_{\sim_{𝔖}})$. This shows that *φ* : [0,1]/~_*𝔖*_ → *ℒ*(*𝔖*) is an epimorphism of lattices. Now, assume that *φ*([*s*]_~_*𝔖*__) = *φ*([*t*]_~_*𝔖*__). Then we deduce that

(18) L(𝔖;s)=ψ𝔖(s)=φ([s]~𝔖)=φ([t]~𝔖)=ψ𝔖(t)=L(𝔖;t),

which implies that *φ* is injective. Hence *φ* is an isomorphism of lattices and so [0,1]^†^/~_*𝔖*_ is isomorphic to $\left(ℒ(𝔖),\tilde{\cup },\tilde{\cap }\right)$.

**Corollary 31**

The lattice [0,1]^†^/~_*𝔖*_ = ([0,1]/~_*𝔖*_, ⊓, ⊔) is a distributive lattice which can be embedded into the lattice $\left({𝒮}_{A}(U),\tilde{\cup },\tilde{\cap }\right)$.

**Proof**

By Corollary 23 $\left({𝒮}_{A}(U),\tilde{\cup },\tilde{\cap }\right)$ is a distributive lattice. Also we know that *ℒ*(*𝔖*) is a sublattice of the lattice $\left({𝒮}_{A}(U),\tilde{\cup },\tilde{\cap }\right)$. Finally, according to Theorem 30, [0,1]^†^/~_*𝔖*_ is isomorphic to the sublattice *ℒ*(*𝔖*). Therefore, [0,1]^†^/~_*𝔖*_ is distributive and can be embedded into the lattice $\left({𝒮}_{A}(U),\tilde{\cup },\tilde{\cap }\right)$.

## 5. Decomposition Theorems of Fuzzy Soft Sets

As mentioned above, the notion of *t*-level soft sets acts as a key factor in solving adjustable fuzzy soft decision-making problems. So it is meaningful to ascertain how many different *t*-level soft sets could be derived from a given fuzzy soft set by choosing distinct threshold value *t* ∈ [0,1]. Motivated by this point, we will explore in this section the problem with regard to the decomposition of a given fuzzy soft set in terms of its *t*-level soft sets.

**Definition 32 (see [26])**

Let $S=(\tilde{F},A)\in ℱ{𝒮}^{E}(U)$. The *value space* of the fuzzy soft set *S* is a set $V(S)={⋃}_{a\in A}\{\tilde{F}(a)(x):x\in U\}$. A scalar *t* ∈ [0,1] is called a *crucial threshold value* of the fuzzy soft set *S* if *t* ∈ *V*(*S*).

Using the above notions, the authors have established the following decomposition theorems of fuzzy soft sets in [26].

**Theorem 33**

Let *S* ∈ *ℱ𝒮* ^*E*^(*U*). Then one has $S={\tilde{{⋃}_{}}}_{t\in [0,1]}t⊙L(S;t)$.

**Theorem 34**

Let *S* ∈ *ℱ𝒮* ^*E*^(*U*). Then one has $S={\tilde{{⋃}_{}}}_{t\in V(S)}t⊙L(S;t)$.

The second decomposition theorem is especially useful when the value space of the fuzzy soft set *S* is finite since in this case we can obtain a finite decomposition of *S*. In order to give a deeper insight into this issue, we propose the following notions.

**Definition 35**

Let *𝔖* ∈ *ℱ𝒮* ^*E*^(*U*) and ~_*𝔖*_ be the level equivalent relation induced by *𝔖*. Then *J*⊆[0,1] is called a *complete threshold set* of *𝔖* if it consists of precisely one element from each equivalence class of [0,1]/~_*𝔖*_.

**Definition 36**

Let *𝔖* ∈ *ℱ𝒮* ^*E*^(*U*) and ~_*𝔖*_ be the level equivalent relation induced by *𝔖*. Then a mapping *f* : [0,1]/~_*𝔖*_ → [0,1] is called a *threshold choice function* of *𝔖* if *f*([*t*]_~_*𝔖*__)∈[*t*]_~_*𝔖*__. The *image* of the threshold choice function *f* is denoted by *Im*(*f*).

In view of the above definitions, we immediately deduce that the image of the threshold choice function *f* is a complete threshold set. It is also evident that, in general cases, both the threshold choice function and the complete threshold set of a given fuzzy soft set *𝔖* might not be unique.

**Proposition 37**

Let *S* be a fuzzy soft set over *U* with a finite value space *V*(*S*) = {*v*_1_,…, *v*_*n*_} such that *v*_1_ < *v*_2_ < ⋯<*v*_*n*_. One has the following.

1. If 1 ∈ *V*(*S*), then [0,1]/~_*S*_ = {[0, *v*_1_], (*v*_1_, *v*_2_],…, (*v*_*n*−1_, *v*_*n*_]}.
2. If 1 ∉ *V*(*S*), then [0,1]/~_*S*_ = {[0, *v*_1_], (*v*_1_, *v*_2_],…, (*v*_*n*−1_, *v*_*n*_], (*v*_*n*_, 1]}.

**Proof**

First, we assume that *v*_*n*_ < 1. For *t* ∈ [0,1], we have the following cases.

- Case 1: *L*(*S*; *t*) = *L*(*S*; *v*_1_) = *𝔘*_*A*_⇔*t*~_*S*_ *v*_1_⇔*t* ∈ [0, *v*_1_].
- Case 2: *L*(*S*; *t*) = *L*(*S*; *v*_*i*_)⇔*t*~_*S*_ *v*_*i*_⇔*t* ∈ (*v*_*i*−1_, *v*_*i*_], (*i* = 2,…, *n*).
- Case 3: *L*(*S*; *t*) = *L*(*S*; 1) = Φ_*A*_⇔*t*~_*S*_1⇔*t* ∈ (*v*_*n*_, 1].

Thus we obtain that

(19) $$\begin{array}{c} \frac{\left[0,1\right]}{\sim_{S}}=\left\{\left[0,v_{1}\right],\left(v_{1},v_{2}\right],\ldots ,\left(v_{n-1},v_{n}\right],\left(v_{n},1\right]\right\}. \end{array}$$

Note also that if 1 ∈ *V*(*S*), then *v*_*n*_ = 1 and (*v*_*n*_, 1] = *∅* should be removed in the above equality (Case 3 simply does not arise). Therefore, it follows that

(20) $$\begin{array}{c} \frac{\left[0,1\right]}{\sim_{S}}=\left\{\left[0,v_{1}\right],\left(v_{1},v_{2}\right],\ldots ,\left(v_{n-1},v_{n}\right]\right\} \end{array}$$

holds when *v*_*n*_ = 1. This completes the proof.

**Remark 38**

The above statement gives an explicit description of the structure of the lattice [0,1]^†^/~_*S*_ = ([0,1]/~_*S*_, ⊓, ⊔) discussed in Theorem 30 and Corollary 31. It says that the level equivalent relation ~_*S*_ divides the unit interval [0,1] into some subintervals and these subintervals actually form a distributive lattice under certain operations.

**Corollary 39**

Let *S* be a fuzzy soft set over *U* with a finite value space *V*(*S*). Then *V*(*S*)∪{1} is a complete threshold set of *S*.

**Proof**

Let *S* be a fuzzy soft set over *U* with a finite value space *V*(*S*) = {*v*_1_,…, *v*_*n*_} such that *v*_1_ < *v*_2_ < ⋯<*v*_*n*_. First, we assume that *v*_*n*_ < 1. By Proposition 37, we have

(21) $$\begin{array}{c} \frac{\left[0,1\right]}{\sim_{S}}=\left\{\left[0,v_{1}\right],\left(v_{1},v_{2}\right],\ldots ,\left(v_{n-1},v_{n}\right],\left(v_{n},1\right]\right\}. \end{array}$$

Let *f* : [0,1]/~_*S*_ → [0,1] be a mapping given by *f*([*t*]_~_*S*__) = ∨[*t*]_~_*S*__. Then *f* is a threshold choice function of the fuzzy soft set *S*, since every subinterval in [0,1]/~_*S*_ contains its least upper bound. It follows that *Im*(*f*) = *V*(*S*)∪{1} is a complete threshold set of *S*.

On the other hand, if *v*_*n*_ = 1 ∈ *V*(*S*), then by Proposition 37, we have

(22) $$\begin{array}{c} \frac{\left[0,1\right]}{\sim_{S}}=\left\{\left[0,v_{1}\right],\left(v_{1},v_{2}\right],\ldots ,\left(v_{n-1},v_{n}\right]\right\}. \end{array}$$

Using similar augments as above, we can show that *Im*(*f*) = *V*(*S*) is a complete threshold set of *S*. But it is clear that *V*(*S*) = *V*(*S*)∪{1}, since *v*_*n*_ = 1. Hence, *V*(*S*)∪{1} is a complete threshold set of *S*.

The following statement explicitly characterizes the structure of the lattice *ℒ*(*S*), consisting of all level soft sets of a fuzzy soft set *S*. By Theorem 30, the structure of *ℒ*(*S*) is closely related to that of the lattice ([0,1]/~_*S*_, ⊓, ⊔) as described in Proposition 37.

**Theorem 40**

Let $S=(\tilde{F},A)$ be a fuzzy soft set over *U* with a finite value space *V*(*S*) = {*v*_1_,…, *v*_*n*_} such that *v*_1_ < *v*_2_ < ⋯<*v*_*n*_. One has the following.

(1) If 1 ∈ *V*(*S*), then the lattice *ℒ*(*S*) is a finite ascending chain as follows:
  (23) $$\begin{array}{c} L\left(S;1\right)=L\left(S;v_{n}\right){\tilde{\subseteq }}_{F}\cdots {\tilde{\subseteq }}_{F}L\left(S;v_{2}\right){\tilde{\subseteq }}_{F}L\left(S;v_{1}\right)={𝔘}_{A} \end{array}$$
(2) If 1 ∉ *V*(*S*), then the lattice *ℒ*(*S*) is a finite ascending chain as follows:
  (24) $$\begin{array}{c} \Phi_{A}=L\left(S;1\right){\tilde{\subseteq }}_{F}L\left(S;v_{n}\right){\tilde{\subseteq }}_{F}\cdots {\tilde{\subseteq }}_{F}L\left(S;v_{2}\right){\tilde{\subseteq }}_{F}L\left(S;v_{1}\right)={𝔘}_{A}. \end{array}$$

**Proof**

Define a mapping *φ* : [0,1]/~_*S*_ → *ℒ*(*S*) by

(25) $$\begin{array}{c} \varphi \left({\left[t\right]}_{\sim_{S}}\right)=L\left(S;\vee {\left[t\right]}_{\sim_{S}}\right). \end{array}$$

By Theorem 30, the mapping *φ* is an isomorphism of lattices. Then the above result follows from Proposition 37 and its proof.

**Theorem 41**

Let $S=(\tilde{F},A)$ be a fuzzy soft set over *U* with a finite value space *V*(*S*) and let *J*⊆[0,1] be a finite set. Then one has

(26) $$\begin{array}{c} S=\tilde{\underset{t\in J}{⋃}\;}t⊙L\left(S;t\right)⟺V\left(S\right)\subseteq J. \end{array}$$

**Proof**

Let ${\tilde{{⋃}_{}}}_{t\in J}t⊙L(S;t)=(\tilde{G},A)$. First, we assume that *V*(*S*)⊆*J*. Then by Theorems 33 and 34, we have

(27) $$\begin{array}{c} S=\tilde{\underset{t\in V(S)}{⋃}\;}t⊙L\left(S;t\right){\tilde{\subseteq }}_{F}\left(\tilde{G},A\right),\\ \left(\tilde{G},A\right){\tilde{\subseteq }}_{F}\tilde{\underset{t\in [0,1]}{⋃}\;}t⊙L\left(S;t\right)=S. \end{array}$$

It follows that $S=(\tilde{G},A)={\tilde{{⋃}_{}}}_{t\in J}t⊙L(S;t)$.

Conversely, suppose that

(28) $$\begin{array}{c} \left(\tilde{G},A\right)=\tilde{\underset{t\in J}{⋃}\;}t⊙L\left(S;t\right)=S=\left(\tilde{F},A\right). \end{array}$$

If *V*(*S*)⊈*J*, then there exists a crucial threshold value $\tilde{F}(a^{*})(u^{*})=t^{*}\in V(S)\setminus J$ for some a* ∈ *A* and u* ∈ *U*. Now, we write the finite set *J* as a disjoint union; namely, *J* = *J* ^−^  ⨄  *J* ^+^, where *J* ^−^ = {*r* ∈ *J* : *r* < t*} and *J* ^+^ = {*r* ∈ *J* : *r* ≥ t*}. Using similar techniques and notations as in the proof of Theorem 34, we deduce

(29) G~(a∗)(u∗) =⋁t∈JG~t(a)(x)=⋁t∈JtFt(a)(x) =(⋁t∈J−t∧Ft(a)(x))∨(⋁t∈J+t∧Ft(a)(x)) =(⋁t∈J−t∧1)∨(⋁t∈J+t∧0)=⋁t∈J−t<t∗=F~(a∗)(u∗).

This contradicts to the hypothesis $\left(\tilde{G},A)=(\tilde{F},A\right)$. Hence *V*(*S*)⊆*J*.

**Remark 42**

The above result shows that the value space *V*(*S*) is the least threshold set for decomposing a fuzzy soft set *S*. In this case, we can find that *V*(*S*) is of crucial importance since we cannot decompose a fuzzy soft set correctly if any of its crucial threshold values is missing. This justifies the term “crucial threshold values” (see Definition 32) for these scalars.

By means of scalar uniproducts proposed in the previous section, we can further obtain the following decomposition theorem.

**Theorem 43**

Let *S* be a fuzzy soft set over *U* with a finite value space *V*(*S*) = {*v*_1_,…, *v*_*n*_} such that *v*_1_ < *v*_2_ < ⋯<*v*_*n*_. Then one has

(30) $$\begin{array}{c} S=\tilde{\underset{2\leq i\leq n}{⋃}}\left(v_{i-1},v_{i}\right]{⊙}_{\cup }L\left(S;v_{i}\right)\tilde{⋃}\left[0,v_{1}\right]{⊙}_{\cup }L\left(S;v_{1}\right). \end{array}$$

**Proof**

Using the definition of scalar uniproducts, we have

(31) $$\begin{array}{c} \left[0,v_{1}\right]{⊙}_{\cup }L\left(S;v_{1}\right)=\tilde{\underset{t\in [0,v_{1}]}{⋃}\;}t⊙L\left(S;v_{1}\right)=v_{1}⊙L\left(S;v_{1}\right). \end{array}$$

For 2 ≤ *i* ≤ *n*, it can be seen that

(32) (vi−1,vi]⊙∪L(S;vi) =⋃t∈(vi−1,vi] ~t⊙L(S;vi)=vi⊙L(S;vi).

By Theorem 34, we know that *S* can be decomposed by using its crucial threshold values. That is, $S={\tilde{{⋃}_{}}}_{1\leq i\leq n}v_{i}⊙L(S;v_{i})$. Hence, it follows that

(33) $$\begin{array}{c} S=\tilde{\underset{2\leq i\leq n}{⋃}}\left(v_{i-1},v_{i}\right]{⊙}_{\cup }L\left(S;v_{i}\right)\tilde{⋃}\left[0,v_{1}\right]{⊙}_{\cup }L\left(S;v_{1}\right). \end{array}$$

This completes the proof.

**Corollary 44**

Let *S* be a fuzzy soft set over *U* with a finite value space *V*(*S*) = {*v*_1_,…, *v*_*n*_} such that *v*_1_ < *v*_2_ < ⋯<*v*_*n*_. Then one has

(34) S=⋃2≤i≤n~(vi−1,vi]⊙∪L(S;vi)⋃~[0,v1]⊙∪L(S;v1)×⋃~(vn,1]⊙∪L(S;1).

**Proof**

If 1 ∈ *V*(*S*), then *v*_*n*_ = 1 and (*v*_*n*_, 1] = *∅*. Thus

(35) $$\begin{array}{c} \left(v_{n},1\right]{⊙}_{\cup }L\left(S;1\right)=\emptyset {⊙}_{\cup }L\left(S;1\right)={\tilde{ℭ}}_{A}^{0}. \end{array}$$

Otherwise, *v*_*n*_ < 1 and so we have

(36) $$\begin{array}{c} \tilde{⋃}\left(v_{n},1\right]{⊙}_{\cup }L\left(S;1\right)=\left(v_{n},1\right]{⊙}_{\cup }\Phi_{A}={\tilde{ℭ}}_{A}^{0}. \end{array}$$

Also it is clear that $S=S\;\tilde{\cup }\;{\tilde{ℭ}}_{A}^{0}$. Therefore, we deduce that

(37) S=⋃2≤i≤n~(vi−1,vi]⊙∪L(S;vi)⋃~[0,v1]⊙∪L(S;v1)×⋃~(vn,1]⊙∪L(S;1).

This completes the proof.

The above results reveal that a given fuzzy soft set with a finite value space could be linked to finite number of all its different *t*-level soft sets, which are derived from a partition of the unit interval [0,1] determined by all crucial threshold values of the given fuzzy soft set.

**Example**

Let us reconsider the fuzzy soft set $𝔖=(\tilde{F},A)$ describing “attractive multimedia cell phones” in Example 9. We hope to give a proper classification and rating of these multimedia cell phones. Clearly, the value space of *𝔖* is a finite set

(38) $$\begin{array}{c} V\left(𝔖\right)=\left\{0.2,0.6,0.7,0.9\right\}. \end{array}$$

By Proposition 37, the level equivalent relation ~_*𝔖*_ divides the unit interval [0,1] into some subintervals and these subintervals actually form a distributive lattice. Specifically, we have

(39) $$\begin{array}{c} \frac{\left[0,1\right]}{\sim_{𝔖}}=\left\{\left[0,0.2\right],\left(0.2,0.6\right],\left(0.6,0.7\right],\left(0.7,0.9\right],\left(0.9,1\right]\right\}. \end{array}$$

With regard to the *t*-level soft sets of the fuzzy soft set *𝔖*, we have the following.

- For *v*_1_ = 0.2,  *L*(*𝔖*; *v*_1_) = *𝔘*_*A*_ is the relative whole soft set with respect to the parameter set *A*.
- For *v*_2_ = 0.6,   *L*(*𝔖*; *v*_2_) = *𝔗*_2_ is a soft set with its tabular representation given by Table 2.
- For *v*_3_ = 0.7,   *L*(*𝔖*; *v*_3_) = *𝔗*_3_ is a soft set with its tabular representation given by Table 3.
- For *v*_2_ = 0.9,  *L*(*𝔖*; *v*_4_) = *𝔗*_4_ is a soft set with its tabular representation given by Table 4.
- For *t* ∈ (0.9,1],   *L*(*𝔖*; *t*) = Φ_*A*_ is the relative null soft set with respect to the parameter set *A*.

In addition, by Theorem 40, we also know that the lattice *ℒ*(*𝔖*), consisting of all level soft sets of a fuzzy soft set *𝔖*, is a finite ascending chain as follows:

(40) ΦA=L(𝔖;1)⊆~FL(𝔖;0.9)⊆~FL(𝔖;0.7)⊆~FL(𝔖;0.6)⊆~FL(𝔖;0.2)=𝔘A.

Finally, by Corollary 44, we can obtain the following decomposition:

(41) 𝔖=[0,0.2]⊙∪𝔘A∪~(0.2,0.6]⊙∪𝔗2∪~(0.6,0.7]⊙∪×𝔗3∪~(0.7,0.9]⊙∪𝔗4∪~(0.9,1]⊙∪ΦA.

It reveals that in total there are five distinct level soft sets which are corresponding to the given fuzzy soft set *𝔖* on different segmentations of the unit interval [0,1] determined by all crucial threshold values. For instance, *𝔗*_2_ is the level soft set corresponding to *𝔖* on the subinterval (0.2,0.6]. Using this level soft set, we can get the following classification and rating of all the cell phones under our consideration:

(42) $$\begin{array}{c} \left\{p_{3}\right\}≻\left\{p_{6}\right\}≻\left\{p_{2},p_{4}\right\}≻\left\{p_{1},p_{5}\right\}. \end{array}$$

This means that if we choose any threshold value 0.2 < *t* ≤ 0.6, then all the cell phones can be graded into four classes. Moreover, *p*_3_ turns out to be the most attractive multimedia cell phones, while *p*_1_ and *p*_5_ form the class of least attractive multimedia cell phones.

## 6. Conclusions

We have investigated the decomposition of fuzzy soft sets with finite value spaces. We proposed scalar uniproduct and int-product operations of fuzzy soft sets. We also defined level equivalent relations and investigated some lattice structures associated with level soft sets. It has been shown that the collection *ℒ*(*𝔖*) of all *t*-level soft sets of a given fuzzy soft set *𝔖* forms a sublattice of the lattice $\left({𝒮}_{A}(U),\tilde{\cup },\tilde{\cap }\right)$. In addition, we proved that the quotient structure [0,1]^†^/~_*𝔖*_ = ([0,1]/~_*𝔖*_, ⊓, ⊔) of the unit interval [0,1] induced by level equivalent relations is isomorphic to the lattice *ℒ*(*𝔖*) of *t*-level soft sets and thus could be embedded into the lattice $\left({𝒮}_{A}(U),\tilde{\cup },\tilde{\cap }\right)$. We also introduced crucial threshold values and complete threshold sets. Moreover, we established some decomposition theorems for fuzzy soft sets with finite value spaces and illustrated its possible practical applications with an example concerning the classification and rating of multimedia cell phones. Note also that our results generalize those classical decomposition theorems of fuzzy sets since every fuzzy set can simply be viewed as a fuzzy soft set with only one parameter. To extend this work, one might consider decomposition of fuzzy soft sets based on variable thresholds or other related applications of decomposition theorems of fuzzy soft sets.

## Conflict of Interests

The authors declare that there is no conflict of interests regarding the publication of this paper.

## Figures and Tables

**Table 1:** Tabular representation of the fuzzy soft set $𝔖=(\tilde{F},A)$.

*U*	*e*_2_	*e*_4_	*e*_5_	*e*_6_	*e*_8_
*p*_1_	0.2	0.9	0.6	0.2	0.2
*p*_2_	0.6	0.6	0.2	0.2	0.9
*p*_3_	0.9	0.7	0.9	0.9	0.7
*p*_4_	0.6	0.2	0.2	0.7	0.6
*p*_5_	0.2	0.6	0.2	0.7	0.2
*p*_6_	0.9	0.2	0.7	0.6	0.9

**Table 2:** Tabular representation of the soft set *𝔗*_2_ = *L*(*𝔖*; 0.6).

*U*	*e*_2_	*e*_4_	*e*_5_	*e*_6_	*e*_8_
*p*_1_	0	1	1	0	0
*p*_2_	1	1	0	0	1
*p*_3_	1	1	1	1	1
*p*_4_	1	0	0	1	1
*p*_5_	0	1	0	1	0
*p*_6_	1	0	1	1	1

**Table 3:** Tabular representation of the soft set *𝔗*_3_ = *L*(*𝔖*; 0.7).

*U*	*e*_2_	*e*_4_	*e*_5_	*e*_6_	*e*_8_
*p*_1_	0	1	0	0	0
*p*_2_	0	0	0	0	1
*p*_3_	1	1	1	1	1
*p*_4_	0	0	0	1	0
*p*_5_	0	0	0	1	0
*p*_6_	1	0	1	0	1

**Table 4:** Tabular representation of the soft set *𝔗*_4_ = *L*(*𝔖*; 0.9).

*U*	*e*_2_	*e*_4_	*e*_5_	*e*_6_	*e*_8_
*p*_1_	0	1	0	0	0
*p*_2_	0	0	0	0	1
*p*_3_	1	0	1	1	0
*p*_4_	0	0	0	0	0
*p*_5_	0	0	0	0	0
*p*_6_	1	0	0	0	1
